# Vitamin D status and its association with season, hospital and sepsis mortality in critical illness

**DOI:** 10.1186/cc13790

**Published:** 2014-03-24

**Authors:** Karin Amrein, Paul Zajic, Christian Schnedl, Andreas Waltensdorfer, Sonja Fruhwald, Alexander Holl, Tadeja Urbanic Purkart, Gerit Wünsch, Thomas Valentin, Andrea Grisold, Tatjana Stojakovic, Steven Amrein, Thomas R Pieber, Harald Dobnig

**Affiliations:** 1 Department of Internal Medicine, Division of Endocrinology and Metabolism, Medical University of Graz, Auenbruggerplatz 15, A-8036 Graz, Austria; 2 Department of Anesthesiology and Intensive Care Medicine, Medical University of Graz, Auenbruggerplatz 15, A-8036 Graz, Austria; 3 Department of Neurology, Division of Neurogeriatrics, Medical University of Graz, Auenbruggerplatz 15, A-8036 Graz, Austria; 4 Department of Medical Informatics, Statistics and Documentation, Medical University of Graz, Auenbruggerplatz 15, A-8036 Graz, Austria; 5 Department of Internal Medicine, Division of Infectious Diseases, Medical University of Graz, Auenbruggerplatz 15, A-8036 Graz, Austria; 6 Institute of Hygiene, Microbiology and Environmental Medicine, Medical University of Graz, Auenbruggerplatz 15, A-8036 Graz, Austria; 7 Clinical Institute of Medical and Chemical Laboratory Diagnostics, Medical University of Graz, Auenbruggerplatz 15, A-8036 Graz, Austria; 8 Private Clinic Kreuzschwestern, Kreuzgasse 35, Graz 8010, Austria; 9 Joanneum Research Forschungsgesellschaft mbH, Leonhardstraß, 8010 Graz, Austria

## Abstract

**Introduction:**

Vitamin D plays a key role in immune function. Deficiency may aggravate the incidence and outcome of infectious complications in critically ill patients. We aimed to evaluate the prevalence of vitamin D deficiency and the correlation between serum 25-hydroxyvitamin D (25(OH) D) and hospital mortality, sepsis mortality and blood culture positivity.

**Methods:**

In a single-center retrospective observational study at a tertiary care center in Graz, Austria, 655 surgical and nonsurgical critically ill patients with available 25(OH) D levels hospitalized between September 2008 and May 2010 were included. Cox regression analysis adjusted for age, gender, severity of illness, renal function and inflammatory status was performed. Vitamin D levels were categorized by month-specific tertiles (high, intermediate, low) to reflect seasonal variation of serum 25(OH) D levels.

**Results:**

Overall, the majority of patients were vitamin D deficient (<20 ng/ml; 60.2%) or insufficient (≥20 and <30 ng/dl; 26.3%), with normal 25(OH) D levels (>30 ng/ml) present in only 13.6%. The prevalence of vitamin D deficiency and mean 25(OH) D levels was significantly different in winter compared to summer months (*P* <0.001). Hospital mortality was 20.6% (135 of 655 patients). Adjusted hospital mortality was significantly higher in patients in the low (hazard ratio (HR) 2.05, 95% confidence interval (CI) 1.31 to 3.22) and intermediate (HR 1.92, 95% CI 1.21 to 3.06) compared to the high tertile. Sepsis was identified as cause of death in 20 of 135 deceased patients (14.8%). There was no significant association between 25(OH) D and C-reactive protein (CRP), leukocyte count or procalcitonin levels. In a subgroup analysis (n = 244), blood culture positivity rates did not differ between tertiles (23.1% versus 28.2% versus 17.1%, *P* = 0.361).

**Conclusions:**

Low 25(OH) D status is significantly associated with mortality in the critically ill. Intervention studies are needed to investigate the effect of vitamin D substitution on mortality and sepsis rates in this population.

## Introduction

In critical illness, sepsis is one of the leading causes of death [1,2]. Despite the substantial improvement of clinical outcomes since the introduction of early goal-directed therapy [3], successful treatment remains a great challenge depending mainly on rapid elimination of the responsible microorganism in addition to supportive treatment [1,4]. Nosocomial infections such as ventilator-associated pneumonia, central line-associated bloodstream infection or catheter-associated urinary tract infection are common in critical illness [5] and associated with excessive costs and mortality [6].

The majority of critically ill patients are vitamin D deficient [7-11]. Vitamin D status is a surrogate marker of mobility and outdoor activities because the majority of body stores are derived from solar UVB and only negligible amounts may come from few, especially fortified, dietary sources [12]. In higher (and lower) latitudes, endogenous vitamin D synthesis is severely restricted during the winter months because a more oblique angle means less UVB reaches the earth [12]. Protective clothing, air pollution and sunscreen also impair optimal cutaneous vitamin D production, explaining the high prevalence of vitamin D deficiency found worldwide, even in otherwise healthy subjects [13].

Besides rickets and osteomalacia, the well-known musculoskeletal manifestations of profound vitamin D deficiency, there is now extensive literature on adverse health outcomes in patients with vitamin D deficiency including increased all-cause and cardiovascular mortality, cardiovascular events and impaired immune function [12,14]. In adult and pediatric critically ill patients, low vitamin D levels have been linked to poor outcomes including higher mortality and extended length of stay [15-20]. The largest observational study published to date (n = 2,399) also reported a higher rate of blood culture positivity in a subgroup with available blood culture results (n = 1,160) [17]. In a small (n = 81), but highly relevant study, patients hospitalized for suspected infection with a baseline 25-hydroxyvitamin D (25(OH) D) level <30 ng/ml had more severe sepsis and all patients who died had a 25(OH) D level <30 ng/ml [21].

Most cell types, including many immune cells such as macrophages, B and T cells, have a nuclear vitamin D receptor (VDR), respond to the active metabolite 1,25-dihydroxyvitamin D (1,25(OH)2D) and possess the enzyme 1α-hydroxylase, which is required to activate native vitamin D to its major metabolite 1,25(OH)2D, responsible for autocrine or paracrine function [22,23]. Vitamin D seems to be essential in both the innate and the adaptive immune system for optimal function of antimicrobial activity [24,25]. Thus, there is a strong biological rationale that vitamin D is not only a surrogate marker of disease severity but also a contributing factor to increased sepsis incidence and severity [11,26]. We therefore analyzed mortality and available clinical data on infectious diseases in a large, retrospective data set of mixed critically ill patients to allow for hypothesis generation and sample size calculation for a planned randomized controlled trial (RCT) at the same study site. We postulated that low 25(OH) D levels are associated with adverse outcomes including blood culture positivity, all-cause and sepsis mortality in an ICU setting.

## Materials and methods

This was a retrospective observational study. The ethics committee at the Medical University of Graz approved the study before data retrieval and waived the requirement for informed consent because the investigators devised procedures to protect the confidentiality of the information to be collected. The primary endpoint was the prevalence of vitamin D deficiency and insufficiency. Secondary endpoints included the association of ICU mortality and hospital mortality with 25(OH) D status, adjusted for covariates like baseline Simplified Acute Physiology Score II (SAPS II), age, gender, glomerular filtration rate (GFR) and C-reactive protein (CRP). A subgroup analysis was performed in patients with available blood culture results during hospital stay. 25(OH) D was measured from fresh blood samples on workdays with enzyme immunoassay (Immunodiagnostic Systems, Boldon, UK) until June 2009 and with the IDS-iSYS, an assay based on chemiluminescence technology (Immunodiagnostic Systems), thereafter (R^2^ 0.93 at our laboratory). The coefficient of variation for control material is 13% at 12 ng/ml, 10% at 31 ng/ml, and 9% at 64 ng/ml [26], respectively. Samples were taken in the early morning along with other routine laboratory tests. In the majority of patients, 25(OH) D was analysed within 48 hours following ICU admission.

### Study sites

The study was conducted at the University Medical Center of Graz, Austria, a 1,559-bed tertiary care facility providing medical care for the province of Styria and its capital city Graz, Austria (47° degrees latitude). Patients admitted to one of the intensive care units treating adult patients between September 2008 and May 2010 were included in this study. The ICUs include a medical, neurological, cardiothoracic surgery and several mixed surgery units with eight to fifteen beds.

### Data retrieval

Adult patients were identified from the hospital’s routine data management system by the Institute of Informatics, Statistics and Documentation. Routine data gathered during hospital stay, including cause of death (during hospital stay) was extracted from the hospital’s medical documentation system. Sepsis-associated mortality was categorized by two physicians independently based upon detailed review of nonsurvivors’ hospital charts using the 2001 international sepsis definitions conference’s publication [27].

### Study population

A total of 655 unique patient data sets with at least one available 25(OH) D level measured during ICU stay were retrieved. In case of multiple results (or ICU stays) only the first measurement (or ICU stay) was taken into account for analysis. The investigation time span had to be limited after May 2010 because we started an intervention study at the same ICUs [28].

### Statistical analysis

Data were analyzed using SPSS Version 20.0 (IBM Corp., Armonk, NY, USA) and are expressed as mean ± standard deviation or median (interquartile range) as appropriate. Between-group comparisons were carried out using chi-square test or Kruskal-Wallis test as appropriate. For survival analyses, Cox regression analyses adjusted for SAPS II, age, gender, GFR and CRP and the log-rank test was used. There was no ethnicity difference in the population studied. The time variable for Cox regression analysis was time to death or hospital discharge. Power analysis was performed extrapolating data from other studies [18,20]. We assumed that in-hospital mortality would be 10% higher among critically ill patients with 25(OH) D <20 ng/ml compared to those with 25(OH) D >20 ng/mL. With an alpha error level of 5% and a sample size of 394 patients with 25(OH) D <20 ng/ml and 261 patients with 25(OH) D >20 ng/mL, the power to detect a difference in in-hospital mortality was 88%. To determine the optimal 25(OH) D cut point for hospital mortality, we utilized the Youden Index [29-31]. It was also analyzed for each season (winter: December, January, February; spring: March, April, May; summer; June, July, August; fall: September, October, November). For this analysis, STATA 10.0MP (StataCorp, College Station, TX, USA) was used.

### Definition of vitamin D deficiency

25(OH) D is considered the best available parameter for the definition vitamin D status [32], because it reflects vitamin D body stores better than other vitamin D metabolites. To date, consensus on optimal vitamin D levels and the definition of vitamin D deficiency exists neither in the general population nor in critically ill patients. Therefore, in our study, we used the definition recommended by the Endocrine Society for at-risk populations (25(OH) D ≤ 19.9 ng/ml deficiency, 20 to 29.9 ng/ml insufficiency) in addition to month-specific tertiles in order to reflect seasonal variability [33] and to evaluate if the associations became stronger in this model.

### Vitamin D supplementation

Due to the retrospective nature of the study, detailed information on vitamin D supplementation was not available. It is current clinical practice at our institution to supplement intravenous multivitamin preparations containing 200 to 220 IU of ergocalciferol or cholecalciferol once a day. As demonstrated in the placebo group of our pilot study [34], this dose does not influence 25(OH) D levels in serial measurements.

### Blood cultures

During hospital stay, blood cultures were taken in 244 patients (37% of the study population). Results were retrieved from two laboratories: the Institute of Hygiene and the Section of Infectious Diseases of the Department of Internal Medicine. All results were thoroughly reviewed for clinical validity by two specialized physicians otherwise uninvolved in routine care of the study population. Contaminants such as coagulase-negative staphylococcus were not considered as positive results.

## Results

Clinical and biochemical characteristics of the population are given in Table 1. Primary reasons for ICU admission were neurologic (157, 30.2%), cardiac surgery (78, 15.0%) and cardiovascular events (62, 11.9%). Other causes included respiratory disease (42, 8.1%), trauma (30, 5.8%), brain surgery (26, 5.0%) and infectious diseases, including sepsis at admission (23, 4.4%). The mean 25(OH) D level was 19.6 ± 11.3 ng/ml. Vitamin D tertiles were calculated for each calendar month, based upon 25(OH) D analysis (median overall 25(OH) D levels: 9.7 vs. 16.3 vs. 28.0 ng/ml, *P* <0.001). The highest level was 79.4 and the lowest 1.3 ng/ml. The majority of all patients was vitamin D deficient (<20 ng/ml; 60.2%) or insufficient (≥20 and <30 ng/dl; 26.3%), while normal 25(OH) D levels (>30 ng/ml) were present in only 13.6% of the population. There were small, but significant differences between mean 25(OH) D levels with regard to type of ICU (mixed surgical 17.6 ± 10.4 ng/ml, medical, 18.6 ± 10.3 ng/ml, cardiothoracic 19.6 ± 11.9, neurologic 22.9 ± 12.3, *P* <0.001). Also, the prevalence of vitamin D deficiency differed between ICUs (mixed surgical 70%, medical 64%, cardiothoracic 63%, neurologic 44%, *P* <0.001).

**Table 1:** General patient characteristics and comparison between survivors and nonsurvivors

	**Overall (n = 655)**	**Survivors (n = 520)**	**Nonsurvivors (n = 135)**
Gender, n (%)			
female	243 (37.1%)	192 (36.9%)	51 (37.8%)
male	412 (62.9%)	328 (63.1%)	84 (62.2%)
Age (years), median (iqr)	65 (22)	64 (23)	69 (20)*
SAPS II, median (iqr)	26 (16)	24 (15)	32 (19)*
Type of ICU, n (%)			
medical	238 (36.3%)	178 (34.2%)	60 (44.4%)
neurologic	164 (25.0%)	147 (28.3%)	17 (12.6%)
mixed surgical	150 (22.9%)	112 (21.5%)	38 (28.1%)
cardiothoracic	103 (15.7%)	83 (16.0%)	20 (14.8%)
Length of ICU stay (days), median (iqr)	6.0 (8.31)	5.1 (7.5)	9.1 (10.7)*
Length of hospital stay (days), median (iqr)	14.9 (18.6)	15.9 (19.1)	11.9 (14.1)*
25(OH) D (ng/ml), median (iqr)	17.3 (13.9)	17.9 (14.1)	13.3 (11.4)*
25(OH) D category, n (% of total population)			
<20 ng/ml	394 (60.2%)	298 (57.3%)	96 (71.1%)
<12 ng/ml	193 (29.5%)	139 (26.7%)	54 (40.0%)
20-30 ng/ml	172 (26.3%)	148 (28.5%)	24 (17.8%)
>30 ng/ml	89 (13.6%)	74 (14.2%)	15 (11.1%)
30-40 ng/ml	50 (7.6%)	42 (8.1%)	8 (5.9%)
40-50 ng/ml	22 (3.4%)	19 (3.7%)	3 (2.2%)
>50 ng/ml	17 (2.6%)	13 (2.5%)	4 (3.0%)
GFR (ml/min), mean ± sd	83 ± 54	88 ± 54	67 ± 50*
CRP (mg/dl), mean ± sd	102 ± 102	89 ± 93	150 ± 118*
Total calcium (mmol/l), mean ± sd	2.12 ± 0.19	2.14 ± 0.18	2.06 ± 0.20*
Ionized calcium (mmol/l), mean ± sd	1.10 ± 0.11	1.11 ± 0.10	1.06 ± 0.12*
Phosphate (mmol/l), mean ± sd	1.18 ± 0.44	1.15 ± 0.39	1.27 ± 0.58*
PTH (pg/ml), median (iqr)	48 (45)	47 (40)	60 (78)*

*Refers to a statistically significant difference between survivors and nonsurvivors (*P* <0.05). PTH, phosphate and ionized/total calcium were available in 598, 649 and 202/596 patients, respectively. 25(OH) D, 25-hydroxyvitamin D; CRP, C-reactive protein; GFR, glomerular filtration rate; iqr, interquartile range; PTH, parathyroid hormone; SAPS II, Simplified Acute Physiology Score II; sd, standard deviation.

In survivors, total/ionized calcium levels were significantly higher and 25(OH) D/PTH/phosphorus levels significantly lower compared to nonsurvivors (Table 1). Some of these parameters also showed significant differences between vitamin D groups (Table 2).

**Table 2:** Comparison between patients with vitamin D deficiency, insufficiency, sufficiency and month specific tertiles

	**Deficiency (n = 394)**	**Insufficiency (n = 172)**	**Sufficiency (n = 89)**	**Low tertile (n = 216)**	**Intermediate tertile (n = 219)**	**High tertile (n = 220)**
Gender, male (%)	61.4%	62.2%	70.8%	59.7%	64.8%	64.1%
Age (years), median (iqr)	65 (22)	65 (23)	65 (25)	68 (22)	64 (26)	64 (23)*
SAPS II, median (iqr)	27 (17)	24 (14)	24 (14)*	30 (16)	24 (15)	24 (15)*
Type of ICU, n (%)						
medical	38.6%	32.6%	33.7%*	42.1%	36.1%	30.9%*
neurologic	18.3%	33.1%	39.3%*	18.5%	21.9%	34.5%*
mixed surgical	26.6%	18.0%	15.7%*	25.0%	23.7%	20.0%*
cardiothoracic	16.5%	16.3%	11.2%*	14.4%	18.3%	14.5%*
25(OH) D (ng/ml), median (iqr)	12.2 (5.2)	24.4 (4.7)	37.5 (12.9)*	9.7 (3.7)	16.3 (8.4)	28.0 (12.4)*
GFR (ml/min), mean ± sd	85±62	81±38	82±40	83±65	84±55	84±39
CRP (mg/dl), mean ± sd	107±95	82±95	117±132*	110±93	100±98	94±112*
Total calcium (mmol/l), mean ± sd	2.11±0.19	2.15±0.18	2.15±0.15*	2.10±0.19	2.12±0.19	2.16±0.16*
Ionized calcium (mmol/l), mean ± sd	1.09±0.12	1.12±0.10	1.10±0.08	1.09±0.12	1.09±0.11	1.12±0.09
Phosphate (mmol/l), mean ± sd	1.19±0.46	1.16±0.37	1.15±0.45	1.22±0.49	1.16±0.42	1.15±0.41
PTH (pg/ml), median (iqr)	52 (55)	47 (34)	45 (36)	54 (66)	49 (45)	43 (32)*

*Refers to a statistically significant difference between subgroups (*P* <0.05). 25(OH) D, 25-hydroxyvitamin D; CRP, C-reactive protein; GFR, glomerular filtration rate; iqr, interquartile range; PTH, parathyroid hormone; SAPS II, Simplified Acute Physiology Score II.

### Season

**Figure 1:**
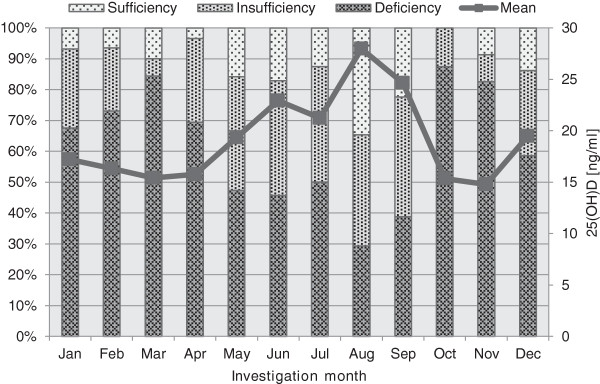
**Seasonal variation of the prevalence of vitamin D deficiency and mean 25(OH) D values.** The prevalence of vitamin D deficiency was higher in winter compared to the summer/autumn months. In August, only 29% were classified as vitamin D deficient, while in or after winter months, the prevalence reached more than 80% (March, 84%, October 87% and November 83%). 25(OH) D, 25-hydroxyvitamin D.

**Figure 2:**
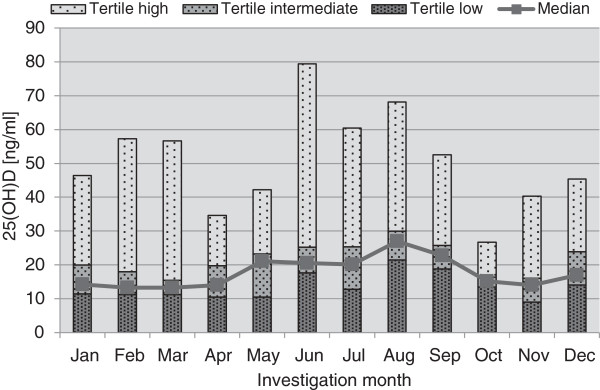
**Seasonal cutoff levels and mean 25(OH) D values for month-specific tertiles.** The highest mean value was observed in August (n = 75, 28.0 ± 13.9 ng/ml). This was significantly and almost two-fold higher than the lowest mean level found in March (n = 90, 15.4 ± 8.4 ng/ml, *P* <0.001), October (n = 16, 15.3 ± 4.9 ng/ml, *P* <0.001) and November (n = 23, 14.8 ± 9.0 ng/ml, *P* <0.001). 25(OH) D, 25-hydroxyvitamin D.

We observed a clear seasonal variation in 25(OH) D serum levels, with the highest mean values observed in August (n = 75, 28.0 ± 13.9 ng/ml). This was significantly and almost two-fold higher than the lowest mean levels found in March (n = 90, 15.4 ± 8.4 ng/ml, *P* <0.001), October (n = 16, 15.3 ± 4.9 ng/ml, *P* <0.001) and November (n = 23, 14.8 ± 9.0 ng/ml, *P* <0.001). The prevalence of vitamin D deficiency was also substantially higher in winter compared to the summer/autumn months. In August, only 29% were classified as vitamin D deficient, while in or after winter months, the prevalence reached more than 80% (March, 84%, October 87% and November 83%) (Figures 1 and 2). The optimal 25(OH) D cut point for hospital mortality was 15.2 ng/ml. This was not season dependent (winter 15.1 ng/ml, spring 15.2 ng/ml, summer 14.9 ng/ml, fall 15.2 ng/ml).

### Mortality

**Figure 3:**
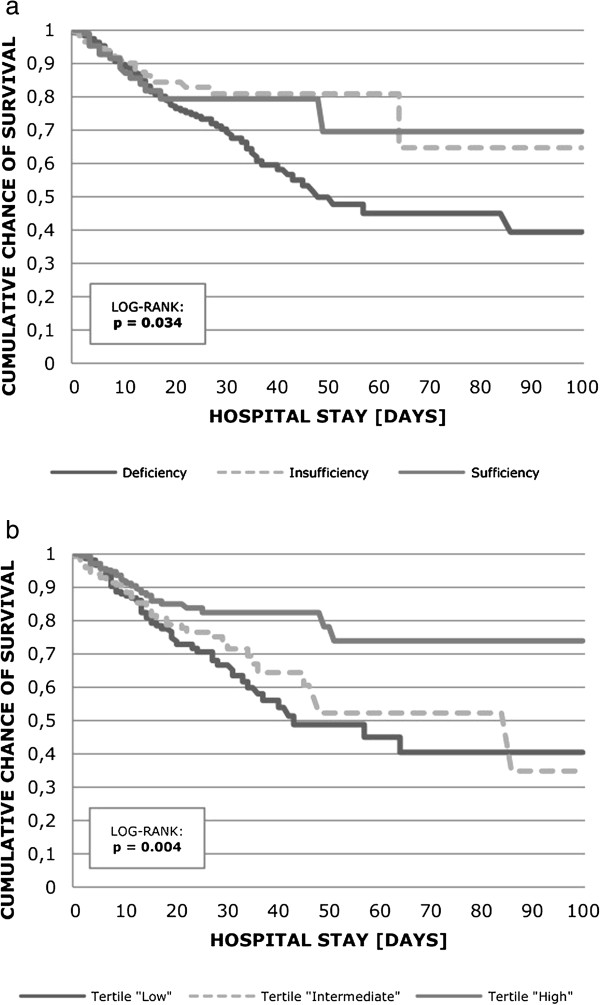
**Unadjusted Kaplan-Meier plot for hospital survival stratified by definition of vitamin D deficiency (a) and month-specific vitamin D tertiles (b).** Using the log-rank test, hospital mortality was significantly different between vitamin D sufficiency, insufficiency and deficiency (*P* = 0.034) and vitamin D tertiles (*P* = 0.004).

All-cause hospital mortality was significantly higher in patients with vitamin D deficiency compared to patients with insufficient and sufficient levels, and the results were even more distinct when hospital mortality rates were compared between month-specific 25(OH) D tertiles (Table 2, Figure 3). In adjusted Cox regression analysis, hazard ratios (HR) were 1.63, 95% confidence interval (CI) 0.93 to 2.86 in vitamin deficiency and 1.01, 95% CI 0.52 to 1.96 in vitamin D insufficiency respectively. For the more specific 25(OH) D tertiles, however, HRs were 2.05, 95% CI 1.31 to 3.22 in the low and 1.92, 95% CI 1.21 to 3.06 in the intermediate tertile respectively. In Cox regression analysis using 25(OH) D as a continuous variable, the HR for hospital mortality was 0.979 (95% CI 0.962 to 0.997).

**Figure 4:**
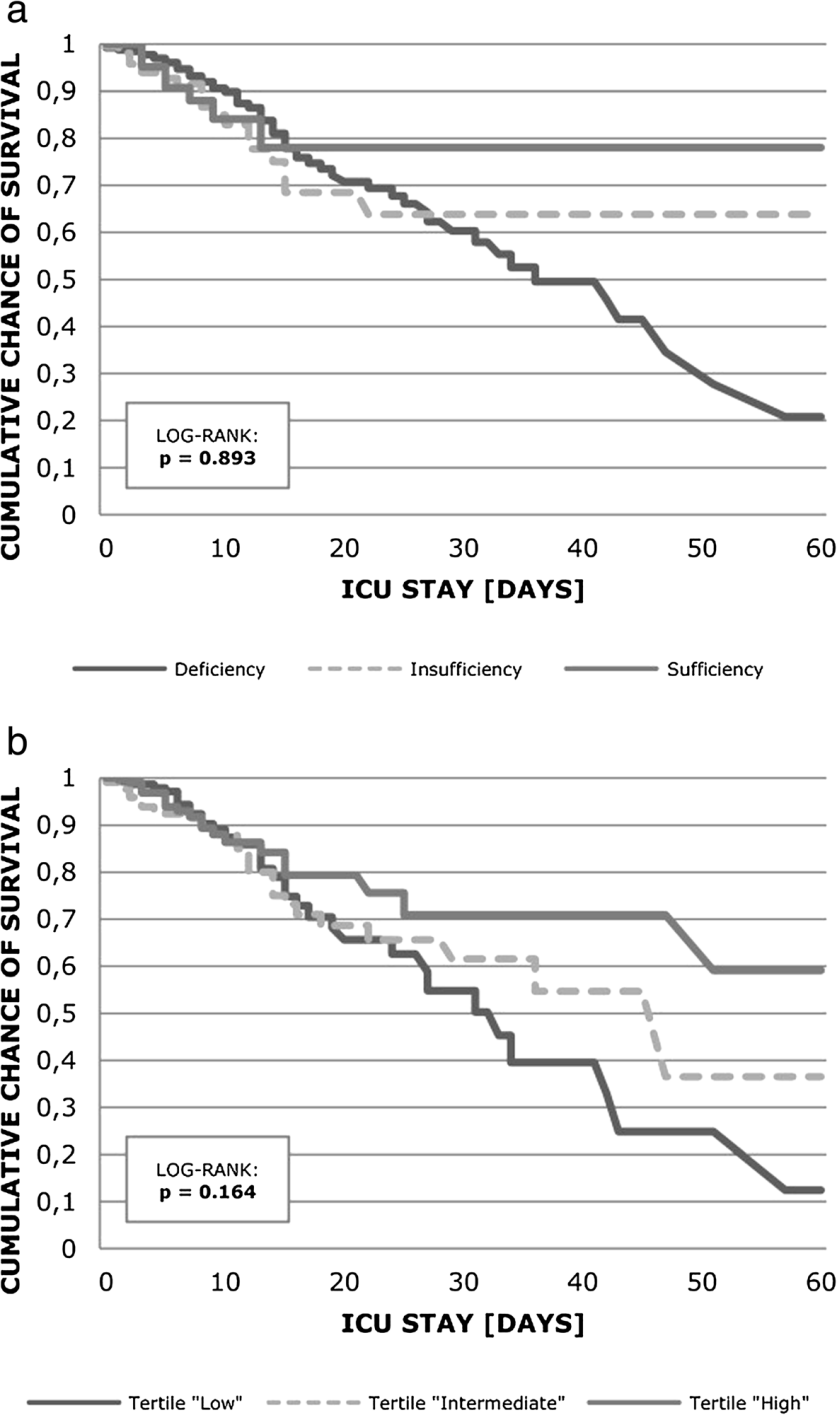
**Unadjusted Kaplan-Meier plot for ICU survival stratified by definition of vitamin D deficiency (a) and month-specific vitamin D tertiles (b).** Using the log-rank test, ICU mortality was not significantly different between groups (*P* = 0.893 for vitamin D sufficiency/insufficiency/deficiency classification and 0.164 for tertiles).

ICU mortality also varied between the three groups, being only numerically higher in both the deficiency and insufficiency groups compared to patients with vitamin D sufficiency. This was also true for the low and intermediate tertile comparisons with the highest tertile (Table 2, Figure 4). HRs were 1.30, 95% CI 0.61 to 2.75 and 1.61, 95% CI 0.70 to 3.67 for deficiency and insufficiency, respectively or 1.70, 95% CI 0.98 to 2.96 and 1.6, 95% CI 0.95 to 2.91 for the low and the intermediate tertile, respectively. In Cox regression analysis using 25(OH) D as a continuous variable, the HR for ICU mortality was 0.997 (95% CI 0.975 to 1.018).

When survival analysis was performed after categorization of vitamin D status into severe deficiency (<12 ng/ml), deficiency (12 to 20 ng/ml), insufficiency (20 to 30 ng/ml) and multiple strata of sufficiency (30 to 40 ng/ml, 40 to 50 ng/ml, >50 ng/ml), ICU mortality was highest in severe deficiency (18.7%) and lowest in the 30 to 40 ng/ml (6.0%) and the 40 to 50 ng/ml (9.1%) groups. Patients with 25(OH) D >50 ng/ml had nonsignificantly higher ICU mortality (17.6%). Hospital mortality was also highest in severe deficiency (28.0%), lowest between 20 and 50 ng/ml (14.0%, 16.0%, 13.6% respectively) and significantly higher in >50 ng/ml (23.5%; *P* = 0.029). In adjusted Cox regression for ICU mortality using 30 to 40 ng/ml as a reference, HRs (95% CI) were as follows: <12 ng/ml: 2.77 (0.85 to 9.10), 12 to 20 ng/ml: 1.80 (0.54 to 6.08), 20 to 30 ng/ml: 2.86 (0.85 to 9.67), 40 to 50 ng/ml: 2.16 (0.34 to 13.59) and >50 ng/ml: 5.07 (1.00 to 25.77). Results for hospital mortality were similar with a HR of 2.12 (1.00 to 4.50) for severe deficiency, 1.64 (0.76 to 3.52) for deficiency, 1.17 (0.52 to 2.64) for insufficiency, 1.20 (0.31 to 4.64) for 40 to 50 ng/ml and 1.66 (0.48 to 5.74) for >50 ng/ml.

### ICU and hospital length of stay

We observed a statistically significant association between vitamin D deficiency and ICU length of stay (LOS) and a trend toward longer ICU stay in the low month-specific tertile. However, there was no association between hospital LOS and vitamin D status (Table 3). In survivors, median ICU LOS was 6.1 days in the deficiency group, 4.4 days in the insufficiency group and 4.7 days in the sufficiency group (*P* = 0.070) or 5.9, 6.0 and 4.9 days in the low, intermediate and high tertiles (*P* = 0.281). Median hospital LOS was 15.8, 16.8 and 15.0 days in the deficiency, insufficiency and sufficiency group (*P* = 0.982) or 14.8, 16.9 and 15.9 days (*P* = 0.840) in the tertiles.

**Table 3:** Descriptive comparison of ICU, hospital and sepsis mortality rates by χ2-test and median ICU and hospital length of stay (LOS) analyzed by Kruskal-Wallis test

	**n**	**ICU mortality**		**Hospital mortality**		**Sepsis mortality**		**ICU LOS**		**Hospital LOS**	
		**n (%)**	***P***	**n (%)**	***P***	**n (%)**	***P***	**Median (iqr)**	***P***	**Median (iqr)**	***P***
Overall	655	88 (13.4%)		135 (20.6%)		20 (3.1%)		6.0 (5.1)		14.9 (12.4)	
Vitamin D classification (ng/ml)											
deficiency, <20	394	58 (14.7%)	0.344	96 (24.4%)	**0.012**	19 (4.8%)	**0.005**	6.9 (9.8)	**0.004**	15.1 (19.1)	0.743
insufficiency, 20-30	172	22 (12.8%)		24 (14.0%)		2 (0.6%)		4.9 (7.7)		15.0 (18.7)	
sufficiency, >30	89	8 (9.0%)		15 (16.9%)		0 (0.0%)		5.2 (6.5)		13.5 (16.8)	
Vitamin D month specific tertiles											
low	216	35 (16.2%)	0.064	58 (26.9%)	**0.002**	11 (5.1%)	**0.005**	6.7 (8.5)	0.097	14.2 (19.6)	0.908
intermediate	219	33 (15.1%)		48 (21.9%)		9 (4.1%)		6.7 (9.0)		15.4 (17.4)	
high	220	20 (9.1%)		29 (13.2%)		0 (0.0%)		5.0 (7.7)		14.8 (18.5)	

*P* values refer to differences between subgroups. iqr, interquartile range.

There were no significant differences in categorized causes of death between groups (data not shown).

### Vitamin D, sepsis and blood culture positivity

Sepsis was identified as cause of death in 20 of 135 deceased patients (14.8%). Mean 25(OH) D levels were 12.3 ± 5.0 as opposed to 18.2 ± 11.2 ng/ml in patients who died from other causes (*P* = 0.022). Not a single sepsis-related fatality was observed in the highest month-specific tertile or in vitamin D sufficiency. When adjusted Cox-regression analysis for lethal sepsis was performed with 25(OH) D as continuous parameter, HR for 25(OH) D was 0.903 (95% CI 0.839 to 0.971).

Blood culture results were available for 244 of 655 patients (37.3%). We did not find significant differences in blood culture positivity rates, neither between month-specific tertiles nor between the vitamin D sufficient, insufficient and deficient groups (Table 4).

**Table 4:** **Descriptive subgroup analysis (n = 244): blood culture positivity rates analyzed with χ**^**2**^**-test**

	**n**	**Blood culture positivity**	
		**n (%)**	***P***
Overall	244	55 (22.5%)	
Vitamin D classification (ng/ml)			
deficiency, <20	160	34 (22.2%)	0.641
insufficiency, 20-30	51	14 (29.2%)	
sufficiency, >30	33	7 (21.2%)	
Vitamin D month-specific tertiles			
low	87	20 (23.1%)	0.361
intermediate	82	22 (28.2%)	
high	75	13 (17.1%)	

There was no significant association between 25(OH) D and CRP (R = -0.009, *P* = 0.81), leukocyte count (R = -0.011, *P* = 0.79) or procalcitonin levels (R = 0.022, *P* = 0.679, n = 372).

## Discussion

We present data on the association of 25(OH) D levels and important clinical outcomes in the second largest cohort of critically ill adults reported. The overall prevalence of vitamin D deficiency exceeded 60% and similar percentages were found in all types of ICUs. Low vitamin D levels were independently associated with all-cause hospital mortality and remained a significant predictor of mortality after multivariate adjustment for relevant confounders, confirming previous findings of adequately powered studies in the critically ill [17,18,20,35]. Interestingly, the survival curves between the groups seemed to separate only after two weeks, a fact for which our data are unable to offer an explanation.

In our cohort, all patients who died from sepsis had 25(OH) D levels <30 ng/ml. This confirms the recent finding of a small study in which sepsis severity was significantly associated with a poor vitamin D status and where all deaths had occurred in patients with 25(OH) D levels <30 ng/ml [21]. In a mixed population of 196 critically ill patients followed up for 28 days, vitamin D deficiency was also associated with a trend toward more infections [36]. Moromizato and colleagues recently showed that a low 25(OH) D level obtained before hospital admission is a significant predictor of sepsis in the critically ill and that these patients have an increased mortality risk [37].

In our study, the risk for blood culture positivity was not significantly associated with vitamin D levels in the subgroup of patients with available blood culture results (n = 244), a fact that may be attributable to small sample size, as in the largest ICU cohort reported to date, Braun *et al*. found a significantly higher rate of positive blood cultures (odds ratio 1.64, 95% CI 1.05 to 2.55, *P* = 0.03) in patients with a 25(OH) D level of <15 ng/ml (subgroup: n = 1160).

In contrast to previous reports [19,38], there was no clear association between ICU or hospital LOS and low vitamin D status in our population. It is of interest, however, that ICU LOS was longer in nonsurvivors compared to survivors.

In the context of discussing clinical outcomes in dependency of 25(OH) D levels, reverse causality needs to be especially considered in the ICU setting. Here, several factors such as fluid loading, acute inflammatory states and lower vitamin D binding protein may affect 25(OH) D levels [39-41]. We are unable to ultimately confirm or reject such observations because details on fluid intakes or vitamin D binding protein levels are unavailable for our cohort. However, in our Cox analysis we included a simultaneous CRP level as a measure of the intensity of the systemic inflammatory response. This is important because a low vitamin D level during hospitalization might only reflect greater degree of inflammation, which would likely be associated with greater mortality. The fact that results remained significant with inclusion of CRP in the model suggests that vitamin D levels are truly *per se* associated with adverse outcomes.

There is a large body of data supporting the concept that vitamin D plays a decisive role in both the innate and the adaptive immune system [24,25,42]. Vitamin D also upregulates the production of important antimicrobial peptides such as β-defensins and cathelicidins that are expressed in barrier tissues like airway, bladder and gastrointestinal epithelium, which are active against many bacteria and fungi, thus protecting the individual from hospital-acquired infection at these important potential entry sites [43]. In critically ill patients, a significant positive correlation between cathelicidin and 25(OH) D serum levels has been reported and cathelicidin levels were lower compared to healthy controls [10]. In a prospective cohort of 112 hospitalized patients with community-acquired pneumonia, neither cathelicidin nor β-defensin-2 levels correlated with 25(OH) D status, but there was a trend toward increased mortality with lower cathelicidin levels [44]. Furthermore, severe vitamin D deficiency (<12 ng/ml) was associated with an OR of 12.7 (95% CI, 2.2 to 73.3) for the 30-day mortality compared to patients with 25(OH) D levels above 20 ng/ml [44]. In an elegant Swedish study using bladder biopsies from healthy postmenopausal women, 2,000 IU of cholecalciferol daily significantly increased cathelicidin production after infection with *Escherichia coli* associated with a direct antibacterial effect [45]. In summary, our data are in line with hypotheses suggesting that vitamin D status is an important contributor to sepsis incidence and outcome [11,46-48].

Another relevant finding of our study is that vitamin D status was surprisingly dependent on season with a maximum difference of a factor of two between the lowest levels in or after winter and the highest levels in August. In other populations, vitamin D status is dependent on season as well [49-52], however, it may be expected that critically ill patients are much less affected by this change because they are often severely restricted in their mobility even before acute illness so that the cutaneously produced amount of vitamin D may be very limited. Our results clearly demonstrate that critically ill patients are not exempt from this general rule with even the magnitude of variation being similar to healthy individuals [51]. Interestingly, the strong seasonal variation closely resembles the described seasonal variation of respiratory tract infections, invasive pneumococcal disease and sepsis [53,54]. Of note, the optimal 25(OH) D cut point for hospital mortality was not season dependent at 15 ng/ml.

In a large cohort of cardiac surgical patients (n = 4,418), Zittermann and colleagues recently demonstrated a significantly increased risk of major cardiac and cerebrovascular events (defined as in-hospital death, myocardial infarction, low output syndrome and stroke) in patients with low (<20 ng/ml), but also high-normal vitamin D status (>40 ng/ml) [55]. Similar results were observed for mechanical ventilation, ICU and hospital stay. The reasons for this finding are unclear to date. At 25(OH) D levels >50 ng/ml, we also observed a higher risk for hospital mortality in our population of mixed critically ill patients. Obviously, this analysis was limited by its small sample size (n = 17, or 2.6% of all patients), but requires special future consideration, as levels in this range are certainly not considered toxic and do not cause hypercalcemia [56]. Such a correlation might also be attributable to vitamin D supplementation in previously deficient patients who are more likely to have a compromised health status.

### Limitations

The most important limitation of this study is its retrospective observational single-center design, which precludes conclusions regarding causality, and selection bias may be present. Although this is one of the largest published cohorts on this topic, it is still of a relatively small sample size for the detection of less pronounced effects so we had insufficient statistical power to detect weaker associations. Unfortunately, for logistic reasons it was impossible to collect data on patients who did not have 25(OH) D measured. Based on the number of patients who stayed in the ICUs in the year before data collection and the collection time frame of 20 months, we have data available for approximately 23% of patients staying >48 hours in ICU and, while 25(OH) D levels were available only in a minority, it was nevertheless a substantially larger fraction compared to other retrospective studies on this topic that included 2 to 5% of the treated population.

Due to data limitations, we are also unable to provide details on 1,25(OH)2D levels, premorbid health status and acute or chronic liver failure, the use of vitamin D supplements, the type of infection and detailed data on ICU morbidity such as organ dysfunction or the duration or intensity of mechanical respiratory or inotropic support. Often critically ill patients have lower albumin, liver dysfunction and therefore lower vitamin D binding protein, which is associated with lower 25(OH) D levels that are still considered to be normal when free levels are normal.

The accuracy of 25(OH) D assays may be influenced by a variety of factors [57,58] and in critical illness this is further complicated by a number of specific patient-related factors [39,58]. The used assays are not as accurate as the expensive gold standard liquid chromatography/tandem mass spectrometry [41], but we used fresh blood samples for 25(OH) D analysis, which was performed on a daily basis in our institution and the used assays are currently widely available tests for routine use. Finally, our study was performed in a Caucasian population in a European academic tertiary care center and may, therefore, be limited in its generalizability.

## Conclusions

Our data demonstrate that low vitamin D levels are highly prevalent in critical illness and linked to higher all-cause and sepsis mortality independent of other important contributors. These findings are important because vitamin D deficiency is a condition that may be easily treated. Compared to other adjunctive interventions used in critical care in general and in sepsis in particular, vitamin D supplementation is a simple, inexpensive and safe therapy. In a pilot study, we have previously shown that a high dose of oral cholecalciferol (540,000 IU loading dose) is able to correct vitamin D deficiency rapidly in most patients [34]. However, adequately powered prospective RCTs are urgently required to evaluate the effect of vitamin D substitution on mortality, sepsis rates and other clinically relevant outcomes in critically ill patients.

## Key messages

• 25(OH) D deficiency is common across different ICUs.

• Vitamin D status exhibits a strong seasonal variation.

• 25(OH) D status is significantly associated with all-cause hospital mortality. The lowest mortality was apparent between 20 and 40 ng/ml.

• Lethal sepsis occurred only in patients with 25(OH) D levels <30 ng/ml.

• The optimal 25(OH) D cut point for hospital mortality was 15 ng/ml.

## Abbreviations

1,25(OH)2D: 1,25-hydroxyvitamin D; 25(OH) D: 25-hydroxyvitamin D; CRP: C-reactive protein; GFR: glomerular filtration rate; ICU: intensive care unit; LOS: length of stay; RCT: randomized controlled trial; SAPS II: Simplified Acute Physiology Score II.w

## Competing interests

KA, CS, AH, TS, TRP and HD are investigators in the VITdAL@ICU trial - a completed vitamin D intervention trial (Clinical Trials Identifier NCT01130181) that is funded by the Austrian National Bank (Jubiläumsfonds, Project Nr. 14143), a grant from the European Society for Clinical Nutrition and Metabolism (ESPEN) and an institutional, unconditional and nonrestrictive research grant from Fresenius Kabi, Austria in addition to the supply of study medication. All other authors declare that they have no competing interests.

## Authors’ contributions

KA, TRP and HD designed the study. GW, TV, TS, AG assisted with data retrieval. KA, PZ and CS performed the statistical analysis and drafted the manuscript. AW, SF, AH, TUP and SA participated in interpreting the data. All authors revised the manuscript carefully for important intellectual content and approved the final version.
